# High-precision isotopic analysis sheds new light on mercury metabolism in long-finned pilot whales (*Globicephala melas*)

**DOI:** 10.1038/s41598-019-43825-z

**Published:** 2019-05-13

**Authors:** Eduardo Bolea-Fernandez, Ana Rua-Ibarz, Eva M. Krupp, Jörg Feldmann, Frank Vanhaecke

**Affiliations:** 1 Ghent University, Department of Chemistry, Atomic & Mass Spectrometry – A&MS research group, Campus Sterre, Krijgslaan 281-S12, 9000 Ghent, Belgium; 2 University of Aberdeen, Department of Chemistry, Trace Element Speciation Laboratory, Meston Walk, Aberdeen, AB24 3UE UK

**Keywords:** Marine mammals, Stable isotope analysis, Element cycles, Mass spectrometry

## Abstract

Whales accumulate mercury (Hg), but do not seem to show immediate evidence of toxic effects. Analysis of different tissues (liver, kidney, muscle) and biofluids (blood, milk) from a pod of stranded long-finned pilot whales (*Globicephala melas*) showed accumulation of Hg as a function of age, with a significant decrease in the MeHg fraction. Isotopic analysis revealed remarkable differences between juvenile and adult whales. During the first period of life, Hg in the liver became isotopically lighter (δ^202^Hg decreased) with a strongly decreasing methylmercury (MeHg) fraction. We suggest this is due to preferential demethylation of MeHg with the lighter Hg isotopes and transport of MeHg to less sensitive organs, such as the muscles. Also changes in diet, with high MeHg intake *in utero* and during lactation, followed by increasing consumption of solid food contribute to this behavior. Interestingly, this trend in δ^202^Hg is reversed for livers of adult whales (increasing δ^202^Hg value), accompanied by a progressive decrease of δ^202^Hg in muscle at older ages. These total Hg (THg) isotopic trends suggest changes in the Hg metabolism of the long-finned pilot whales, development of (a) detoxification mechanism(s) (*e.g*., though the formation of HgSe particles), and Hg redistribution across the different organs.

## Introduction

Mercury (Hg) is a toxic heavy metal that is globally distributed through the atmosphere, in which Hg has a residence time of up to 1 year^1,2^. Atmospheric Hg is predominantly deposited in oceans, one of the major reservoirs of Hg on Earth^3^. In aquatic environments, inorganic Hg species are converted into the more toxic compound methylmercury (MeHg). This conversion proceeds *via* biotic or abiotic methylation, occurring in sediments and/or in the water column^4^. MeHg exposure of aquatic biota occurs mainly *via* the diet, resulting in the bioaccumulation and biomagnification of MeHg across food webs, leading to high Hg levels in predatory animals^5^.

Located at the top of aquatic food chains and with a long lifespan, marine mammals accumulate high amounts of Hg in their tissues^6,7^. This is an issue of great concern in terms of seafood safety, as despite of the health risks, these marine species are consumed by humans at some locations around the world^8^. However, the study of marine mammals is even more relevant owing to the similarities these species may share with humans in terms of metabolic pathways. It has been shown that despite the high Hg concentrations present in their tissues, marine mammals do not show the toxic effects observed for humans, suggesting the existence of (an) effective Hg detoxification mechanism(s)^9^. Thus, study of the Hg metabolic pathways (*i.e*., uptake, distribution and excretion) in marine mammals may aid (*i*) our understanding of the behavior of Hg in humans and thus, (*ii*) the development of new strategies for avoiding and/or minimizing the toxic effects induced by Hg and its compounds, such as neurological damage and cardiovascular problems.

Some authors previously reported a species-specific Hg distribution among the different organs of marine mammals^10,11^, and pointed out the affinity of Hg for biomolecules containing S and Se. The covalent binding of Hg to thiol/sulfhydryl groups is a well-known biochemical mechanism, involved in transport of Hg across membranes, its redistribution across different tissue types and its excretion^1,12,13^. However, there is increasing evidence that the physiological target of Hg is not S, but Se (binding of Hg to selenoproteins and selenoenzymes)^14,15^. In fact, the binding affinity between Hg and Se is several orders of magnitude higher than that between Hg and S. Thus, Se may play a more important role than S in Hg detoxification, especially *via* the formation of insoluble, stable and inert Hg-Se bonds, *e.g*., in HgSe particles^16–19^. However, it needs to be noted that several studies also suggested the incorporation of S into the HgSe structures^20–22^. In any case, important knowledge gaps still remain in our understanding of the metabolic pathways of Hg in marine mammals.

The measurement of Hg isotope ratios is considered a promising approach to shed light onto the biogeochemical Hg cycle in nature^23,24^. Hg is one of the few elements that display not only mass-dependent isotope fractionation (MDF; typically expressed *via* the δ^202^Hg value), but also mass-independent isotope fractionation (MIF; expressed using the Δ^199^Hg and Δ^201^Hg values), thus enabling a multi-dimensional approach^25,26^. The occurrence of MDF has been documented for most of the transformations that Hg undergoes in the environment, including methylation, demethylation, ligand exchange, adsorption, volatilization, liquid-vapor evaporation, abiotic oxidation and reduction^27–35^. Additionally, MIF of a large magnitude has been observed during photochemical reactions involving radicals (see Methods for the definition of δ^202^Hg, Δ^199^Hg and Δ^201^Hg)^36^. Several laboratory experiments and field studies have indicated that trophic transfer of Hg and most of the *in vivo* processes it is involved in are accompanied by *in vivo* MDF^11,37^. However, *in vivo* MIF is not observed for these processes, suggesting that the MIF signature of Hg – Δ^199^Hg, Δ^201^Hg and Δ^199^Hg/Δ^201^Hg – in aquatic animals (especially those located at the top of a trophic chain) is typically more useful for provenance determination of the accumulated Hg than the MDF signature^36,38–41^.

In this work, different tissues and biofluids (liver, kidney, muscle, blood and milk) of long-finned pilot whales *(Globicephala melas)* were analyzed for their bulk or total Hg (THg) concentration, MeHg concentration and THg isotope ratios. Our aim was to (*i*) obtain a more profound insight into the metabolic processes that Hg undergoes in these marine mammals and to (*ii*) find evidence for effective detoxification mechanisms avoiding Hg poisoning.

## Sample Collection and Sample Types

On the 12^th^ of September 2012, 31 long-finned pilot whales (*Globicephala melas*) stranded on a beach between Ansturther and Pittenween in Scotland, United Kingdom. From the complete pod, 10 whales were refloated, but 21 whales died at the stranding site, where the autopsy of each animal was carried out and their organs were dissected and stored for research purposes, as part of the government stranding scheme, aiming to establish the reason behind Cetaceous strandings on the Scottish coast^9^. We have analyzed 73 samples, comprising 21 liver samples, 20 kidney samples, 15 muscle samples, 14 blood samples and 1 milk sample. In addition, HgSe particles were isolated from 5 liver samples and 2 muscle samples, and subsequently characterized in terms of Hg isotopic composition. The age of the whales was determined by Gajdosechova *et al*.^9^ following the method described by Lockyer^42^. Supplementary Table 1 provides further information (age, gender and length) on the animals analyzed in this work.

## Mercury Distribution in Long-Finned Pilot Whales

**Figure 1:**
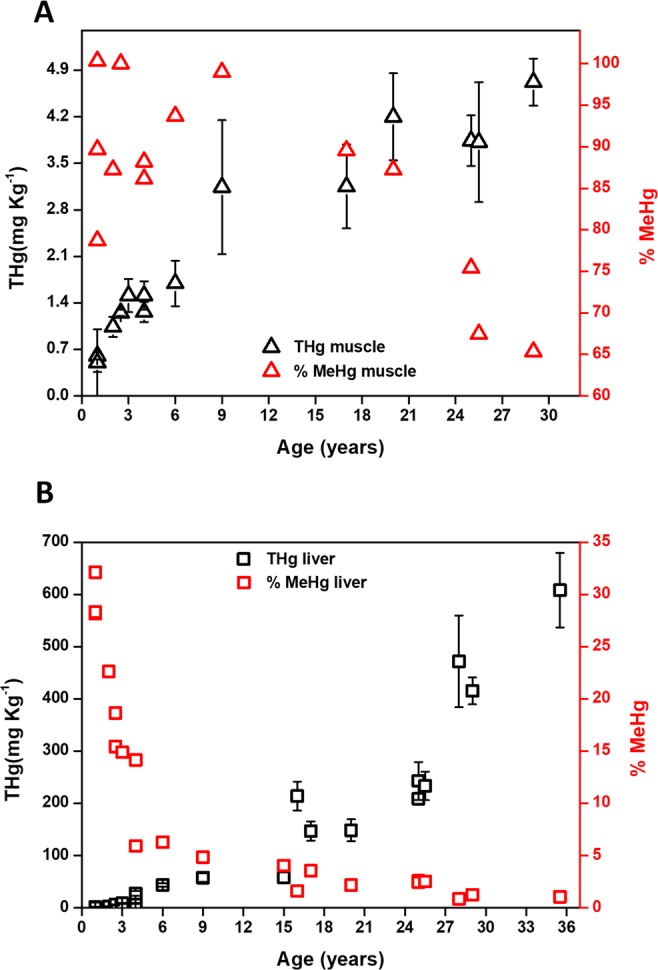
THg concentration (black, in mg Kg^−1^ w.w.) and % MeHg (red) as a function of age for muscle (**A**) and liver (**B**) tissues of long-finned pilot whales. The error bars correspond with the SD of three digestion replicates.

For every individual analyzed, the highest level of Hg was found in liver (0.98–608 mg Kg^−1^ wet weight, w.w.), followed by kidney (0.42–21.8 mg Kg^−1^ w.w.) and muscle (0.50–4.72 mg Kg^−1^ w.w.) (see Supplementary Tables 2–4 for THg concentrations and MeHg fractions). This THg concentration trend is in good agreement with data previously reported for marine mammals^6,9^. Speciation analysis indicated that Hg is predominantly present as MeHg in muscle (65.3–100%), while the MeHg fraction in liver (1.0–32.1%) and kidney (4.7–46.5%) tends to be relatively low. Based on the assumption that the Hg is mainly taken in by marine mammals from the diet (including transport across the placenta before birth and *via* milk though lactating)^43^, the relatively low MeHg fraction observed in liver and kidney suggests that the MeHg ingested is slowly demethylated *in vivo* and converted into inorganic Hg (iHg) forms. This iHg is stored in the liver, and to a lesser extent in the kidney (also a small fraction of the iHg might be excreted from the organism), while the remaining MeHg is mainly accumulated in the muscle, which acts as a reservoir for this highly toxic Hg species^11,41^. For all tissues studied, the THg concentration increases as a function of age, while the MeHg fraction decreases (Fig. 1). This suggests that the Hg metabolism changes over the lifespan of the whales, most likely inducing a higher extent of MeHg demethylation and/or a greater accumulation of iHg, *e.g*., under the form of less toxic HgSe particles^10,11^.

Also isotopic analysis of THg (Supplementary Fig. 1 shows the three-isotope plots) revealed important differences between the different tissues studied (see Supplementary Tables 2–4 for δ^199^Hg, δ^200^Hg, δ^201^Hg, δ^202^Hg, Δ^199^Hg and Δ^201^Hg values for liver, kidney and muscle, respectively). Low δ^202^Hg values (a lighter isotopic composition) are found for liver (−1.23–−0.15‰) and kidney (−1.10–−0.29‰) tissues, and higher ones for muscle (0.20–1.31‰). These differences can be broadly attributed to the compound-specificity of the Hg isotopic signatures, *i.e*., the different Hg isotopic compositions of MeHg and iHg (Supplementary Fig. 2 shows the δ^202^Hg value as a function of MeHg fraction in muscle and liver) – see next section for further details on specific trends. This could theoretically stem from either different MeHg and iHg sources with different Hg isotopic signatures and/or from metabolic processes occurring in the whale body, such as MeHg demethylation, and the isotope fractionation accompanying these processes. However, the constant Δ^199^Hg and Δ^201^Hg values obtained for the different tissues (average values of 1.05 ± 0.05 and 0.87 ± 0.05‰, respectively) suggest strongly that (*i*) there is no *in vivo* MIF accompanying the different Hg metabolic processes, and that (*ii*) both Hg compounds have the same origin, *i.e*., MeHg ingested *via* the diet (see Supplementary Figs 3 and 4). The absence of *in vivo* MIF observed in this work is consistent with previous studies^11,36,39,44,45^. As indicated above, MeHg and the demethylation product iHg are subsequently distributed differently over the organs. IHg is mainly accumulated in the liver and the kidneys, whereas MeHg is preferentially excreted from the liver and subsequently accumulated in muscle tissue^6,11,41^. Therefore, differences in Hg speciation and the corresponding variations in the isotopic composition of Hg in the different tissues most likely result from *in vivo* demethylation of MeHg^46^, and the MDF of Hg accompanying this process (the lighter Hg isotopes are preferentially demethylated), resulting in higher δ^202^Hg values in the remaining MeHg fraction (muscle) and in lower δ^202^Hg values in the iHg produced (liver, kidney)^47^.

## Differences in Mercury Metabolism Between Juvenile and Adult Whales

**Figure 2:**
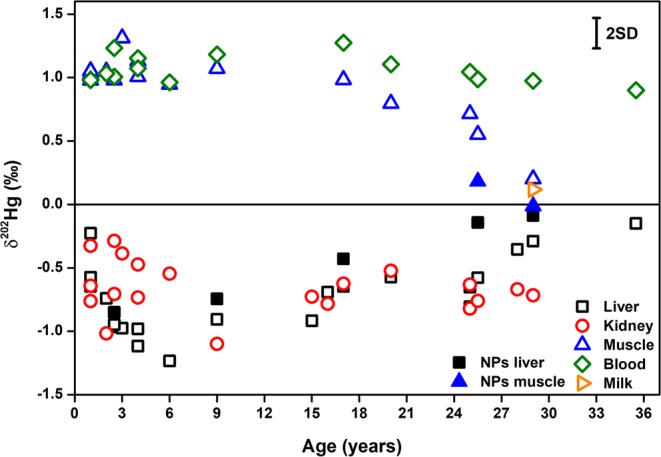
Overview of the MDF-Hg isotopic signatures (δ^202^Hg) of different tissues of long-finned pilot whales as a function of age. The uncertainty reported is the external reproducibility (2SD, n = 147) for the in-house standard.

The combination of MeHg intake and *in vivo* demethylation processes in the whale body explains both the Hg speciation and the bulk Hg isotopic signatures observed in general terms, but additional information is obtained when looking at the results obtained into greater detail. Figure 2 shows the δ^202^Hg values as a function of age for all tissues (muscle, liver and kidney) and biofluids (blood and milk) from female and male individuals (no gender-based differences were observed within the entire study).

### Muscle

**Figure 3:**
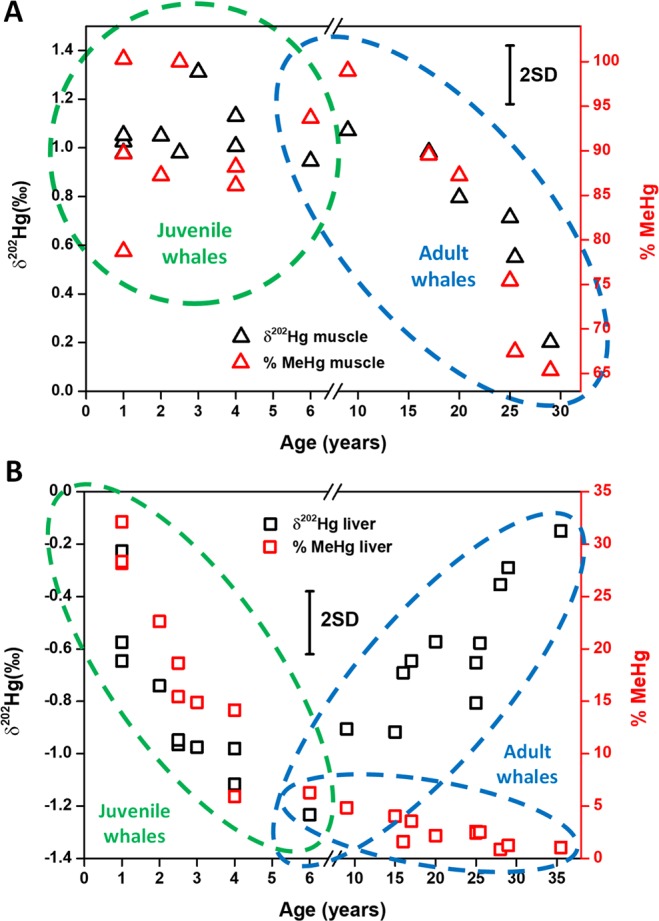
δ^202^Hg (black – left y-axis) and MeHg fraction (% MeHg, red – right y-axis) obtained for muscle (**A**) and liver (**B**) as a function of age. Uncertainty expressed as 2SD of the in-house standard.

We found that muscle tissue is characterized by a high MeHg fraction, and, as a consequence, by a heavier Hg isotopic composition than kidney and liver (a significant positive correlation was found between muscle δ^202^Hg values and MeHg fractions, Spearman’s correlation, r = 0.596, p = 0.025). Recent studies have also reported an enrichment of approximately 1‰ in δ^202^Hg for muscle tissue of seals relative to the Hg isotopic signature of their diet^11^. As can be seen in Fig. 3A, no significant correlations were found between muscle δ^202^Hg values or MeHg fractions and the age of the youngest (≤6 years old) individuals (Spearman’s correlation, r = −0.153, p = 0.694 and r = 0.000, p = 1.00, respectively). It needs to be noted that, within this age interval, additional trends might be hidden by the uncertainty associated with the method used to estimate the ages of younger individuals^42^. For whales ranging between 9 and 29 years old, however, both the δ^202^Hg value and the MeHg fraction decreased systematically in the muscle tissue as a function of the individual’s age: δ^202^Hg is lowered by ~0.9‰ at 29 compared to at 9 years old (Spearman’s correlation, r = −1.000, p = 0.000), while the MeHg fraction in muscle decreased from 99 to 65% MeHg (Spearman’s correlation, r = −1.000, p = 0.000). This was found to be in good agreement with the compound-specificity of the Hg isotopic signatures. The specific trend observed for muscle of adult whales from certain ages onwards suggests metabolic changes in the whale body^48,49^. The redistribution of Hg from highly contaminated organs, such as liver (characterized by low δ^202^Hg values and a high iHg fraction), to muscle might be seen as a protective mechanism against Hg toxicity^50^. In addition, we suggest that muscle loss at older age – the replenishing of muscle is not at the same rate as the loss after a certain age – may be responsible for this variation in the metabolism. The aging muscle is gradually storing more Hg under the form of iHg species, while mobilization of the MeHg stored in this tissue occurs. An example of MeHg mobilization as a function of muscle loss was reported on in literature for migrating birds^51^. During migration, the birds lost an important amount of muscle, inducing a mobilization of MeHg stored in this tissue. The mobilized MeHg can be transported *via* the bloodstream to vital organs, such as brain, where it can cause neurological damage that has hypothetically been identified as the origin of loss of orientation while traveling long distances^52,53^. Next to the redistribution of Hg from contaminated vital organs in adult whales, an increase in the level of MeHg demethylation (assuming preferential demethylation of the lighter Hg isotopes) into less toxic iHg species, *e.g*., under the form of HgSe particles that remain in the muscle tissue, together with the mobilization of MeHg, may explain both the reduction of the MeHg fraction and the lighter bulk Hg isotopic composition of muscle tissues for the oldest whales.

### Liver

In contrast to muscle, the MeHg fraction in liver is generally low, showing a clearly more pronounced decrease for individuals ranging from 1 to 6 years old (see Fig. 3B). At these low ages (≤6 years old), both the δ^202^Hg value and the MeHg fraction decrease following the same pattern (Spearman’s correlation, r = 0.939, p = 0.000), which is in agreement with the enrichment of lighter isotopes in the iHg fraction. The different δ^202^Hg trends are hypothesized to be related with both differences in diet and in Hg metabolism between juvenile and adult whales. Honda *et al*. suggested a higher food intake for younger Antartcic minke whales (*Balaenoptera acutorostrata*) than for older animals^54,55^. Additionally, very low excretion rates by the fetus, specific requirements in newborns and differences in tissues undergoing rapid development may explain the different Hg metabolic pathways of long-finned pilot whales as a function of the individuals’ age (these mechanisms will be discussed into detail below)^6,56–59^.

To assess the influence of MeHg intake from the placenta (*in utero* exposure during gestation) and/or from mother’s milk during lactation on the MeHg fraction and Hg isotopic signature for juvenile whales, the isotopic trend observed for the youngest whales was compared with the Hg isotopic signatures of 14 blood samples and 1 milk sample from a lactating whale (see Supplementary Table 5). In previous studies of dolphins (*Stenella coeruleoalba*, *Sotalia guianensis and Tursiops truncates*), high MeHg exposure *via* the placenta was reported, whereas Hg concentrations in mother milk were found to be higher than those in the prey fish of adult animals^60,61^. Additionally, high MeHg concentrations have been reported for breast milk from lactating women exposed to MeHg *via* the diet^62^, and for rats and mice (Holtzman rat and Balb/c CUM mice)^63,64^. In this work, relatively constant and high δ^202^Hg values (~1‰) were observed for all blood samples (blood can be related with *in utero* exposure), while the milk sample was characterized by a δ^202^Hg value that is significantly lower than that of the blood sample and similar to that of muscle tissue (0.12 ± 0.03‰) from the same whale. Compared to the corresponding blood sample, the Hg isotopic composition of the milk sample was found to be shifted towards the values observed for liver tissues of the youngest whales. Based on these results and on previous studies on MeHg transfer across the placenta and on MeHg in mother’s milk during lactation, we suggest that during the first years of life, the isotopic composition of Hg in the liver is affected by a strong contribution of MeHg originating from the mother, leading to anomalously higher δ^202^Hg values in the liver of juvenile whales. MeHg from the placenta is mainly related with the mother’s blood, characterized by high δ^202^Hg (~1‰), while the introduction of MeHg from lactation already leads to lower δ^202^Hg values (~0.10‰). With the slow introduction of solid food in the diet, δ^202^Hg tends to even lower values. This gradual trend in diet and thus, in MeHg source, may explain the trend observed for the δ^202^Hg value and MeHg fraction in liver tissue of juvenile long-finned pilot whales. It needs to be pointed out that the influence of mother’s milk was based on a single lactating whale only. Nevertheless, the result for that only milk sample available fits within the Hg isotopic data obtained in this work for different tissues and biofluids of long-finned pilot whales and its interpretation was found to be in good agreement with previous hypotheses reported on in literature (see above). Since reliable toxicity data for marine mammals are scarce, the results obtained during this type of post-mortem studies provide valuable information for a better understanding of the complex Hg biochemistry in whales.

In addition to the differences in diet and Hg exposure pathways between juvenile and adult whales indicated above, the influence of detoxification mechanisms developed in an attempt to mitigate the toxic effects of Hg cannot be discarded as a potential explanation for the fast decrease in the MeHg fraction, *i.e*., in comparison with the increase in THg concentration, as a function of age. In previous works, it has been reported that young marine mammals show higher percentages of MeHg than do adult animals, suggesting that they do not reach a specific threshold^65^. Also, it has been hypothesized that young marine mammals are unable to demethylate MeHg efficiently, since the corresponding detoxification mechanism only develops at higher age^66^. Interestingly, a clear and rather abrupt change in the δ^202^Hg *vs* MeHg fraction trend was observed for liver as of an age of approximately 6 years old (Fig. 3B); it needs to be noted that the estimation of complete weaning is 6 years for short-finned pilot whales, thus potentially indicating the development of more efficient detoxification mechanisms to avoid Hg poisoning^67^. As of that age, the decrease in the liver MeHg fraction – from ~6 to 1% for individuals between 5–6 and 35.5 years old – is accompanied by a progressive enrichment in the heavier Hg isotopes (to ~1‰), an anomalous behavior that cannot be explained by the preferential demethylation of lighter Hg isotopes. Masbou *et al*. have very recently reported the same δ^202^Hg trend as a function of the MeHg fraction in livers of beluga whales (*Delphinapterus leucas*)^41^. In that work, the Hg isotopic trend was tentatively explained by factors such as lifetime changes in foraging behavior or ecosystem MDF, as both products from *in vivo* hepatic MeHg breakdown are retained within the whale organism (and within the liver) owing to the lack of other more efficient MeHg excretion pathways, such as those observed in other marine mammals, *e.g*., ringed seals (hair)^68,69^. Perrot *et al*. also suggested that after MeHg demethylation, the product (iHg) and the residual MeHg can be distributed to different organs and tissues by the bloodstream, where they are likely complexed by specific ligands^11^. Unfortunately, none of the works cited above reports on the Hg isotopic composition of muscle tissues as a function of the age. Based on our results, a continuous recirculation of both iHg and MeHg between liver and muscle can most likely explain the trends in the δ^202^Hg values for liver and muscle, as for the oldest whales, the δ^202^Hg values of both tissue types converge towards the same value (see Figs 2 and 3).

In addition to these mechanisms, excretion processes, characterized by preferential removal of lighter Hg isotopes, might also contribute to the trend observed in liver of long-finned pilot whales^45,70^. In previous works based on different species, including fish and humans, it was observed that only a fraction of the iHg generated by MeHg demethylation processes accumulates in liver, while another fraction is excreted from the liver *via* the urine or feces^44,71^. In order to provide further insight into the excretion mechanisms, kidney tissues were studied for their Hg isotopic composition. However, the clear differences in tendency observed for δ^202^Hg in liver between juvenile and adult whales were not found for kidney (see Supplementary Fig. 5).

Also, next to the commonly reported MeHg breakdown resulting in other iHg species, the formation of inert HgSe particles aiming to counteract Hg intoxication has been demonstrated to play an important role in MeHg detoxification, and their formation and associated isotope fractionation need to be taken into account (see next section).

## Evidence for Development of Mercury Detoxification Pathways in Long-Finned Pilot Whales

As it was found that an important fraction of the Hg accumulated in the liver is present under the form of HgSe particles, the formation of these less toxic or even inert particles is assumed to be crucial in Hg detoxification^17,18,66^. Se has a protective effect against Hg toxicity owing to the Hg-Se antagonism^72^. Previous works reported a strong positive correlation between Hg and Se concentrations in muscle tissues of five different species of toothed whales^73^, and in liver, kidney and muscle of long-finned pilot whales^17^. This correlation suggests that Se-mediated MeHg demethylation processes might take place in the whale body. Therefore, the so-called Hg-Se antagonistic effect seems to be responsible for the decrease of the MeHg fraction as a function of age, and for the increase in THg concentrations in liver, kidney and muscle tissues as a result of the formation of less toxic HgSe particles^11,74^.

**Figure 4:**
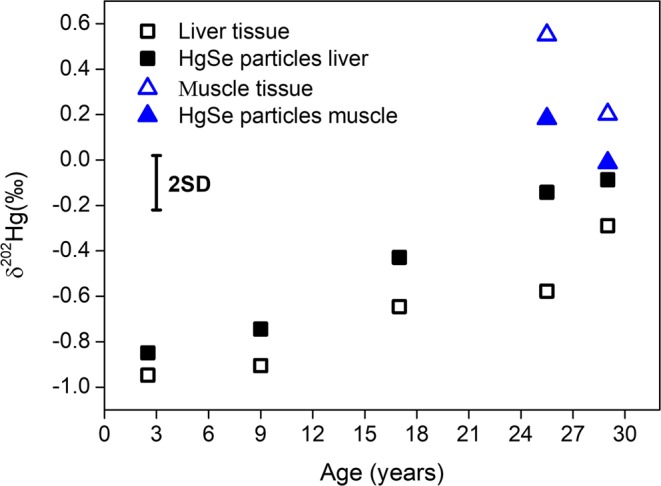
δ^202^Hg values for liver and muscle tissues and for the corresponding isolated HgSe particles. Uncertainty expressed as 2SD of the in-house standard.

According to the mechanisms proposed, the formation of crystalline HgSe is mediated by Hg–metallothionein (Hg-MT) interaction and/or by Hg–Se binding in high-molecular-weight substances^22,75^. These mechanisms have been mainly observed in the liver of marine mammals, stressing the key role of this organ in the biochemistry of Hg. In addition to liver, HgSe particles have also been found in other tissues, such as brain, kidney, lung, muscle, pancreas, pituitary and spleen^17,76,77^. The formation process of HgSe particles in marine mammals is still poorly understood and important knowledge gaps, including the Hg species acting as precursor(s), possible mechanisms and the accompanying Hg isotope fractionation, exist. To obtain further insight, HgSe particles extracted from liver and muscle tissues were analyzed for their Hg isotopic composition (the protocol for extraction and confirmation of the identity of HgSe particles is reported elsewhere)^17^. The results are shown in Supplementary Table 6 and in Fig. 4. δ^202^Hg values of HgSe particles from muscle were found to be lower than that of THg in the corresponding muscle tissue (mostly MeHg), *i.e*., the HgSe particles are enriched in the lighter Hg isotopes. This observation is in agreement with the preferential precipitation of lighter isotopes observed in the formation of metacinnabar (β-HgS) and montroydite (HgO)^78^. In contrast to muscle, δ^202^Hg values of HgSe particles from liver were always higher than that of THg in the corresponding liver tissue. It needs to be stressed that this at first sight opposite trend can be clarified by taking into account that in both tissue types – liver and muscle ─ the THg isotopic composition is a combination (weighted average) of the Hg isotopic compositions of the different Hg species (MeHg and iHg) present. In the case of liver, most of the Hg is in the form of iHg, while only a small fraction of MeHg is present (the percentage of MeHg in liver is a function of the individual’s age). Perrot *et al*.^11^ demonstrated that after MeHg breakdown in the liver, the product iHg is enriched in the lighter Hg isotopes, while the remaining MeHg is enriched in the heavier ones. Therefore, to elucidate the type and extent of fractionation induced by the formation of HgSe particles in the liver (mixture of iHg and MeHg), the Hg species acting as precursor needs to be taking into account. HgSe particles can theoretically form from either iHg compounds resulting from a previous *in vivo* demethylation, or directly from MeHg as part of a detoxification mechanism^16,22^. If we tentatively assume that the same fractionation (direction) is accompanying the formation of HgSe particles in muscle and liver tissues of long-finned pilot whales, then the HgSe particles must stem from MeHg demethylation. The process of HgSe formation from MeHg would then in both tissues be accompanied by isotope fractionation in favor of the lighter isotopes. However, it needs to be pointed out that Hg isotopic analysis of the different Hg species (species-specific Hg isotopic analysis) would be required to experimentally verify this hypothesis and accurately assess the extent of this fractionation. In the absence of species-specific Hg isotopic compositions, the assumption of HgSe particles as a product of MeHg demethylation is supported by (*i*) the formation of HgSe particles as of early ages, although to a lesser extent for juvenile than for adult whales^17^, (*ii*) the lack of mobility of HgSe particles *i.e*., they stay at the “location” of their formation^76,77^, and (*iii*) the occurrence of HgSe particles in muscle tissue of juvenile whales (Hg for ~100% present as MeHg). This explains the differences in δ^202^Hg between the HgSe particles and the corresponding isotopic composition of THg for both tissue types (based on the assumption of a heavier Hg isotopic composition of the MeHg in the liver), although the influence of this process on the δ^202^Hg trends of liver and muscle tissues as a function of age is not self-evident. However, it needs to be noted that the extent of the Hg isotope fractionation induced during HgSe formation (based on the assumption that HgSe particles are a product of MeHg demethylation and on an estimation using isotopic analysis of THg, instead of species-specific isotopic analysis) seems relatively low compared to that accompanying the more “conventional” MeHg breakdown into other iHg species.

We suggest that with the increase in the THg concentration in liver and muscle tissues as a function of age, the formation of other iHg species is increasingly replaced by the formation of HgSe particles. In this case, the relatively low extent of Hg isotope fractionation associated with HgSe formation (see Fig. 4) most likely contributes to the trends observed for the δ^202^Hg value in muscle and liver tissues of adult whales.

Further investigation into the complex Hg biochemistry in marine mammals is still required in order to shed more light onto this complex system. However, this work demonstrates that the information attainable using Hg isotopic analysis may help to increase our understanding of the different Hg metabolic processes occurring during the lifespan of long-finned pilot whales.

## Methods

Samples were taken from 21 pilot whales (*Globicephala melas*) that died upon stranding at a beach between Ansturther and Pittenween in Scotland, United Kingdom. Sampling was carried out under the lead of the Scottish Stranding scheme. In agreement with the Convention on International Trade in Endangered Species of Wild Fauna and Flora (CITES), a European Protected Species license justified the possession and analysis of cetacean samples. All samples (0.2–1.2 g wet weight) and certified reference materials (CRMs – see Supplementary Table 8) used for validation purposes were acid-digested in pre-cleaned closed microwave vessels using a Milestone (Italy) Ethos One High-Performance Microwave Digestion System using a 3:1 mixture of 7 M HNO_3_ and 9.8 M H_2_O_2_. The microwave program is given in Supplementary Table 7. After the digestion, the samples were kept at 4 °C until THg quantification and Hg isotopic analysis.

For speciation (MeHg) analysis, the samples were solubilized in tetramethylammonium hydroxide, buffered with acetate buffer, propylated with sodium tetrapropylborate (NaBPr_4_), extracted into 2,2,4-trimethylpentane and stored at −20 °C until analysis^9,79^.

In addition, HgSe particles were isolated from the corresponding tissues following an enzymatic digestion and their identity was corroborated (>90% HgSe), as reported on in a previous work from Gajdosechova *et al*.^17^. The solubilization of these particles was done by adding a 3:1 mixture of 7 M HNO_3_ and 9.8 M H_2_O_2_.

### Elemental and speciation analysis

Total Hg (THg) concentrations were determined using a ThermoScientific (Germany) Element XR single-collector sector-field ICP-mass spectrometry instrument (SF-ICP-MS) working at low mass resolution, as was previously described by Rua-Ibarz *et al*.^80,81^. For QA/QC of the entire analytical procedure, three CRMs with similar matrix composition as those of the samples were analysed - BCR CRM 464 (tuna fish), NRC-CNRC DORM-4 (fish muscle) and TORT-3 (lobster hepatopancreas) – and the Hg recoveries obtained were between 93–107%.

Hg speciation was performed at the University of Aberdeen using a HP-6890 GC-unit (Agilent Technologies, Japan) coupled to an Agilent 7500 ICP-MS instrument (Agilent Technologies, Japan), as previously described by Gajdosechova *et al*.^9^. The analytical method was validated using two CRMs – NRC-CNRC DOLT-2 and DORM-2. The results obtained were in agreement with the certified values (recoveries between 93–110%).

### Mercury isotopic analysis

Hg isotopic analysis was carried out using a ThermoScientific (Germany) Neptune multi-collector ICP-MS (MC-ICP-MS) instrument equipped with nine Faraday cups. Hg was introduced as Hg(0) generated *via* the reduction of Hg^2+^ with 3% SnCl_2_.2H_2_O in 1.2 M HCl in an HGX-200 cold vapor & hydride generation unit (Teledyne Cetac Technologies, US). The Hg(0)-loaded carrier gas coming from the CVG unit was admixed in a ‘T’ piece with a wet aerosol of Tl (used for internal mass discrimination correction purposes) generated by using a 100 µL min^−1^ concentric nebulizer mounted onto a dual (cyclonic and Scott-type) spray chamber. A complete description of this set-up can be found in a previous manuscript from the same authors^80^.

For instrumental mass discrimination correction, a combination of internal correction using the “Baxter approach” (with NIST SRM 997 Tl as admixed internal standard) and external correction in a sample-standard bracketing (SSB) approach (with NIST SRM 3133 Hg as external standard) was relied on^80,82^.

An in-house standard solution of Hg and the CRMs selected (see Supplementary Table 8) in this work were measured in-between the samples (approximately every five samples) for QA/QC of the measurements and of the entire analytical procedure (*i.e*., MW acid digestion, storage, dilution and subsequent MC-ICP-MS measurement).

The Hg concentration and acid content of all samples, standards and CRMs were matched within ±10% as to avoid inaccurate results. No blank subtraction was applied because the effect of the blank was demonstrated to be negligible within the experimental precision.

The Hg isotopic composition is reported in delta (δ^xxx^Hg) and capital delta (Δ^xxx^Hg) notation – in per mil units (‰) – for mass-dependent (MDF) and mass-independent (MIF) fractionation, respectively^83^.

$${\partial }^{xxx}Hg\,({\rm{\textperthousand }})=\,(\frac{{({}^{xxx}Hg/{}^{198}Hg)}_{sample}}{{({}^{xxx}Hg/{}^{198}Hg)}_{NISTSRM3133}}-1)\ast 1000$$

where xxx = 199, 200, 201 or 202 and NIST SRM 3133 is the Hg isotopic reference material.

$$\begin{array}{rcl}{\rm{\Delta }}{}^{199}Hg & = & \,\partial \,{}^{199}Hg-(\partial {}^{202}Hg\ast 0.2520)\\ {\rm{\Delta }}{}^{200}Hg & = & \partial {}^{200}Hg-\,(\partial {}^{200}Hg\ast 0.5024)\\ {\rm{\Delta }}{}^{201}Hg & = & \partial {}^{201}Hg-\,(\partial {}^{202}Hg\ast 0.7520)\end{array}$$

Following the recommendations of Blum and Bergquist^83^, the analytical uncertainty or long-term external reproducibility of the method has been reported as 2SD of the in-house standard, while for the reference materials, the uncertainty was indicated as 2SE obtained upon replicate analysis (see Supplementary Table 8). The results obtained for the samples analyzed in this work and shown in Supplementary Tables 2–6 are also expressed as average ± 2SE of the measured replicates. For most of the cases, the 2SE values were found to be lower than the external reproducibility of the method, and therefore, the 2SD values of the in-house standard have been reported in all Figures.

## Supplementary information

SUPPLEMENTARY INFORMATION: High-precision isotopic analysis sheds new light on mercury metabolism in long-finned pilot whales (Globicephala melas)
