# Supplementary material for: High-precision isotopic analysis sheds new light on mercury metabolism in long-finned pilot whales (Globicephala melas)
Source: Sci Rep. 2019 May 13;9:7262. doi: 10.1038/s41598-019-43825-z (PMC6513992; doi:10.1038/s41598-019-43825-z)
Supplement: Supplementary file 1 — SUPPLEMENTARY INFORMATION: High-precision isotopic analysis sheds new light on mercury metabolism in long-finned pilot whales (Globicephala melas) [file 41598_2019_43825_MOESM1_ESM.docx]

**SUPPLEMENTARY INFORMATION**

**High-precision isotopic analysis sheds new light on**

**mercury metabolism in long-finned pilot whales (*Globicephala melas*)**

Eduardo Bolea-Fernandez^a**^, Ana Rua-Ibarz^a**^, Eva M. Krupp^b^, Jörg Feldmann^b^, Frank Vanhaecke^a*^

^a^Ghent University, Department of Chemistry, Atomic & Mass Spectrometry – A&MS research group, Campus Sterre, Krijgslaan 281-S12, 9000 Ghent, Belgium

^b^University of Aberdeen, Department of Chemistry, Trace Element Speciation Laboratory, Meston Walk, Aberdeen, AB24 3UE, UK

^*^Correspondence and requests for materials should be addressed to F.V. (email: Frank.Vanhaecke@UGent.be)

^**^Both authors contributed equally to this work.

**Supplementary Table 1.** Information on the pod of whales analyzed in this work

| **Whale ID** | **Age (years)** | **Gender** | **Length (cm)** |
| --- | --- | --- | --- |
| 1 | 2.5 | F | 291 |
| 2 | 25.5 | F | 420 |
| 3 | 17 | F** | 389 |
| 4 | 25* | F** | 420 |
| 5 | 20 | F** | 411 |
| 6 | 1* | F | 192 |
| 7 | 1* | F | 191 |
| 8 | 1* | F | 194 |
| 9 | 6* | M | 333 |
| 10 | 4 | F | 291 |
| 11 | 29 | F** | 445 |
| 12 | 3 | F | 315 |
| 13 | 9 | F | 360 |
| 14 | 2.5 | M | 296 |
| 15 | 35.5 | F | 462 |
| 16 | 4 | M | 318 |
| 17 | 2 | M | 287 |
| 18 | 25 | F | 440 |
| 19 | 15 | M | 444 |
| 20 | 28 | F | 435 |
| 21 | 16 | M | 538 |

*Age estimated based on body length

**Lactating mothers

**Supplementary Table 2.** THg (reported in mg Kg^-1^ w.w.), % MeHg and Hg isotopic composition measured in liver tissue of long-finned pilot whales. The uncertainty is reported as 2SE for three sample replicates.

|  | **Whale ID** | **Age**  **(years)** | **THg**  **(mg Kg^-1^)** | **% MeHg** | **δ^199^Hg**  **(‰)** | **δ^200^Hg**  **(‰)** | **δ^201^Hg**  **(‰)** | **δ^202^Hg**  **(‰)** | **Δ^199^Hg**  **(‰)** | **Δ^201^Hg**  **(‰)** |
| --- | --- | --- | --- | --- | --- | --- | --- | --- | --- | --- |
| **Females** | 6 | 1 | 0.98 ± 0.10 | 32.1 | 0.89 ± 0.05 | -0.32 ± 0.08 | 0.34 ± 0.05 | -0.65 ± 0.12 | 1.05 ± 0.04 | 0.83 ±0.06 |
|  | 7 | 1 | 1.33 ± 0.26 | 28.2 | 0.98 ± 0.06 | -0.29 ± 0.02 | 0.46 ± 0.08 | -0.57 ± 0.04 | 1.12 ± 0.06 | 0.89 ± 0.10 |
|  | 8 | 1 | 0.98 ± 0.15 | 28.3 | 0.89 ± 0.12 | -0.10 ± 0.09 | 0.70 ± 0.07 | -0.23 ± 0.15 | 0.94 ± 0.12 | 0.87 ± 0.05 |
|  | 1 | 2.5 | 5.9 ± 1.0 | 18.6 | 0.81 ± 0.08 | -0.40 ± 0.06 | 0.17 ± 0.06 | -0.97 ± 0.05 | 1.05 ± 0.09 | 0.89 ± 0.07 |
|  | 12 | 3 | 8.7 ± 1.1 | 14.9 | 0.79 ± 0.06 | -0.43 ± 0.04 | 0.18 ± 0.03 | -0.97 ± 0.07 | 1.04 ± 0.06 | 0.91 ± 0.08 |
|  | 10 | 4 | 27.4 ± 4.7 | 5.9 | 0.70 ± 0.05 | -0.55 ± 0.04 | -0.03 ± 0.05 | -1.12 ± 0.03 | 0.98 ± 0.05 | 0.81 ± 0.05 |
|  | 13 | 9 | 57.7 ± 8.2 | 4.8 | 0.78 ± 0.02 | -0.44 ± 0.03 | 0.16 ± 0.08 | -0.91 ± 0.04 | 1.00 ± 0.03 | 0.84 ± 0.08 |
|  | 3 | 17 | 147 ± 18 | 3.6 | 0.88 ± 0.08 | -0.29 ± 0.04 | 0.31 ± 0.04 | -0.65 ± 0.03 | 1.04 ± 0.08 | 0.80 ± 0.06 |
|  | 5 | 20 | 148 ± 21 | 2.2 | 0.92 ± 0.02 | -0.24 ± 0.01 | 0.44 ± 0.07 | -0.57 ± 0.05 | 1.06 ± 0.03 | 0.87 ± 0.06 |
|  | 18 | 25 | 243 ± 36 | 2.6 | 0.82 ± 0.06 | -0.30 ± 0.03 | 0.34 ± 0.09 | -0.65 ± 0.07 | 0.99 ± 0.06 | 0.84 ± 0.05 |
|  | 4 | 25 | 208 ± 10 | 2.4 | 0.73 ± 0.05 | -0.36 ± 0.02 | 0.19 ± 0.07 | -0.81 ±0.09 | 0.93 ± 0.07 | 0.80 ± 0.01 |
|  | 2 | 25.5 | 234 ± 27 | 2.5 | 0.98 ± 0.03 | -0.21 ± 0.03 | 0.49 ± 0.01 | -0.58 ± 0.05 | 1.13 ± 0.02 | 0.93 ± 0.03 |
|  | 20 | 28 | 472 ± 88 | 0.9 | 0.96 ± 0.05 | -0.13 ± 0.07 | 0.55 ± 0.03 | -0.35 ± 0.09 | 1.05 ± 0.03 | 0.81 ± 0.05 |
|  | 11 | 29 | 415 ± 26 | 1.2 | 0.94 ± 0.04 | -0.08 ± 0.04 | 0.66 ± 0.02 | -0.29 ± 0.05 | 1.01 ± 0.04 | 0.87 ± 0.03 |
|  | 15 | 35.5 | 608 ± 71 | 1.0 | 1.01 ± 0.03 | -0.01 ± 0.05 | 0.78 ± 0.03 | -0.15 ± 0.03 | 1.05 ± 0.02 | 0.89 ± 0.04 |
| **Males** | 17 | 2 | 2.61 ± 0.44 | 22.6 | 0.95 ± 0.02 | -0.36 ± 0.03 | 0.39 ± 0.05 | -0.74 ± 0.03 | 1.13 ± 0.03 | 0.95 ± 0.07 |
|  | 14 | 2.5 | 6.14 ± 0.55 | 15.4 | 0.78 ± 0.08 | -0.44 ± 0.10 | 0.10 ± 0.09 | -0.95 ± 0.13 | 1.02 ± 0.05 | 0.82 ± 0.01 |
|  | 16 | 4 | 8.6 ± 1.2 | 14.2 | 0.77 ± 0.05 | -0.47 ± 0.03 | 0.10 ± 0.09 | -0.98 ± 0.04 | 1.02 ± 0.04 | 0.84 ± 0.06 |
|  | 9 | 6 | 43.9 ± 3.5 | 6.3 | 0.71 ± 0.05 | -0.60 ± 0.03 | -0.03 ± 0.11 | -1.23 ± 0.09 | 1.02 ± 0.05 | 0.89 ± 0.09 |
|  | 19 | 15 | 58.2 ± 11 | 4.0 | 0.80 ± 0.06 | -0.45 ± 0.04 | 0.13 ± 0.05 | -0.92 ± 0.07 | 1.03 ± 0.08 | 0.82 ± 0.03 |
|  | 21 | 16 | 214 ± 28 | 1.6 | 0.83 ± 0.05 | -0.33 ± 0.06 | 0.25 ± 0.08 | -0.69 ± 0.05 | 1.00 ± 0.04 | 0.77 ± 0.04 |

**Supplementary Table 3.** THg (reported in mg Kg^-1^ w.w.), % MeHg and Hg isotopic composition measured in kidney tissue of long-finned pilot whales. The uncertainty is reported as 2SE for three sample replicates.

|  | **Whale ID** | **Age**  **(years)** | **THg**  **(mg Kg^-1^)** | **% MeHg** | **δ^199^Hg**  **(‰)** | **δ^200^Hg**  **(‰)** | **δ^201^Hg**  **(‰)** | **δ^202^Hg**  **(‰)** | **Δ^199^Hg**  **(‰)** | **Δ^201^Hg**  **(‰)** |
| --- | --- | --- | --- | --- | --- | --- | --- | --- | --- | --- |
| **Females** | 6 | 1 | 0.60 ± 0.25 | 32.8 | 0.89 ± 0.08 | -0.33 ± 0.09 | 0.32 ± 0.08 | -0.76 ± 0.15 | 1.08 ± 0.05 | 0.89 ± 0.04 |
|  | 7 | 1 | 0.54 ± 0.08 | 41.9 | 0.94 ± 0.09 | -0.33 ± 0.06 | 0.34 ± 0.13 | -0.64 ± 0.13 | 1.11 ± 0.08 | 0.83 ± 0.03 |
|  | 8 | 1 | 0.42 ± 0.09 | 32.2 | 0.92 ± 0.09 | -0.14 ± 0.04 | 0.51 ± 0.06 | -0.33 ± 0.01 | 1.00 ± 0.09 | 0.76 ± 0.07 |
|  | 1 | 2.5 | 1.07 ± 0.10 | 46.5 | 1.00 ± 0.07 | -0.09 ± 0.02 | 0.68 ± 0.12 | -0.29 ± 0.05 | 1.07 ± 0.07 | 0.90 ± 0.11 |
|  | 12 | 3 | 1.31 ± 0.58 | 40.9 | 0.88 ± 0.05 | -0.17 ± 0.05 | 0.52 ± 0.11 | -0.39 ± 0.06 | 0.98 ± 0.04 | 0.81 ± 0.06 |
|  | 10 | 4 | 1.8 ± 1.0 | 33.7 | 0.89 ± 0.05 | -0.24 ± 0.04 | 0.50 ± 0.06 | -0.47 ± 0.04 | 1.01 ± 0.04 | 0.86 ± 0.06 |
|  | 13 | 9 | 6.3 ± 1.3 | 13.2 | 0.77 ± 0.07 | -0.54 ± 0.04 | 0.02 ± 0.08 | -1.10 ± 0.06 | 1.05 ± 0.08 | 0.85 ± 0.04 |
|  | 3 | 17 | 5.15 ± 0.74 | 28.7 | 0.75 ± 0.08 | -0.32 ± 0.02 | 0.31 ± 0.04 | -0.62 ± 0.06 | 0.91 ± 0.09 | 0.78 ± 0.01 |
|  | 5 | 20 | 9.8 ± 3.8 | 10.4 | 0.89 ± 0.04 | -0.24 ± 0.05 | 0.45 ± 0.03 | -0.52 ± 0.08 | 1.02 ± 0.02 | 0.84 ± 0.04 |
|  | 18 | 25 | 15.0 ± 5.5 | 10.5 | 0.83 ± 0.05 | -0.41 ± 0.07 | 0.33 ± 0.11 | -0.82 ± 0.11 | 1.03 ± 0.05 | 0.95 ± 0.04 |
|  | 4 | 25 | 4.31 ± 1.2 | 11.7 | 0.89 ± 0.06 | -0.32 ± 0.04 | 0.25 ± 0.13 | -0.63 ± 0.05 | 1.05 ± 0.07 | 0.73 ± 0.10 |
|  | 2 | 25.5 | 10.6 ± 0.9 | 12.8 | 0.90 ± -0.02 | -0.32 ± 0.03 | 0.29 ± 0.05 | -0.76 ± 0.03 | 1.09 ± 0.03 | 0.86 ± 0.03 |
|  | 20 | 28 | 20.7 ± 1.3 | 4.7 | 0.86 ± 0.03 | -0.33 ± 0.12 | 0.38 ± 0.09 | -0.67 ±0.12 | 1.03 ± 0.01 | 0.88 ± 0.08 |
|  | 11 | 29 | 21.8 ± 2.8 | 7.6 | 0.84 ± 0.03 | -0.33 ± 0.06 | 0.35 ± 0.12 | -0.72 ± 0.05 | 1.02 ± 0.04 | 0.88 ± 0.11 |
|  | 15 | 35.5 | N.A. | N.A. | N.A. | N.A. | N.A. | N.A. | N.A. | N.A. |
| **Males** | 17 | 2 | 1.22 ± 0.21 | 24.6 | 0.76 ± 0.05 | -0.50 ± 0.08 | 0.11 ± 0.14 | -1.02 ± 0.15 | 1.02 ± 0.05 | 0.87 ± 0.06 |
|  | 14 | 2.5 | 1.93 ± 0.44 | 26.1 | 0.85 ± 0.06 | -0.31 ± 0.08 | 0.36 ± 0.08 | -0.71 ± 0.08 | 1.03 ± 0.04 | 0.89 ± 0.03 |
|  | 16 | 4 | 2.99 ± 0.49 | 24.2 | 0.87 ± 0.06 | -0.33 ± 0.01 | 0.34 ± 0.05 | -0.73 ± 0.08 | 1.06 ± 0.08 | 0.89 ± 0.02 |
|  | 9 | 6 | 2.80 ± 0.10 | 34.2 | 0.98 ± 0.02 | -0.25 ±0.06 | 0.46 ± 0.04 | -0.55 ± 0.03 | 1.12 ± 0.02 | 0.87 ± 0.02 |
|  | 19 | 15 | 6.03 ± 0.62 | 26.2 | 0.89 ± 0.02 | -0.35 ± 0.02 | 0.34 ± 0.05 | -0.73 ± 0.06 | 1.07 ± 0.01 | 0.89 ± 0.04 |
|  | 21 | 16 | 7.0 ± 1.4 | 11.1 | 0.85 ± 0.04 | -0.41 ± 0.01 | 0.29 ± 0.07 | -0.78 ± 0.07 | 1.05 ± 0.03 | 0.88 ± 0.05 |

N.A. = not available

**Supplementary Table 4.** THg (reported in mg Kg^-1^ w.w.), % MeHg and Hg isotopic composition measured in muscle tissue of long-finned pilot whales. The uncertainty is reported as 2SE for three sample replicates.

|  | **Whale ID** | **Age**  **(years)** | **THg**  **(mg Kg^-1^)** | **% MeHg** | **δ^199^Hg**  **(‰)** | **δ^200^Hg**  **(‰)** | **δ^201^Hg**  **(‰)** | **δ^202^Hg**  **(‰)** | **Δ^199^Hg**  **(‰)** | **Δ^201^Hg**  **(‰)** |
| --- | --- | --- | --- | --- | --- | --- | --- | --- | --- | --- |
| **Females** | 6 | 1 | 0.51 ± 0.15 | 89.7 | 1.38 ± 0.12 | 0.57 ± 0.11 | 1.70 ± 0.09 | 1.03 ± 0.03 | 1.12 ± 0.13 | 0.93 ± 0.06 |
|  | 7 | 1 | 0.61 ± 0.06 | 100.0 | 1.39 ± 0.06 | 0.59 ± 0.04 | 1.71 ± 0.05 | 1.05 ± 0.11 | 1.13 ± 0.09 | 0.92 ± 0.06 |
|  | 8 | 1 | 0.50 ± 0.09 | 78.7 | 1.23 ± 0.11 | 0.54 ± 0.01 | 1.58 ± 0.02 | 0.98 ± 0.04 | 0.99 ± 0.12 | 0.84 ± 0.05 |
|  | 1 | 2.5 | 0.95 ± 0.05 | 100.0 | 1.28 ± 0.05 | 0.48 ± 0.04 | 1.60 ± 0.03 | 0.98 ± 0.03 | 1.04 ± 0.06 | 0.86 ± 0.04 |
|  | 12 | 3 | 1.51 ± 0.25 | N.A. | 1.37 ± 0.06 | 0.68 ± 0.05 | 1.84 ± 0.07 | 1.31 ± 0.09 | 1.04 ± 0.06 | 0.85 ± 0.03 |
|  | 10 | 4 | 1.51 ± 0.21 | 86.1 | 1.35 ± 0.03 | 0.62 ± 0.04 | 1.72 ± 0.09 | 1.13 ± 0.09 | 1.07 ± 0.04 | 0.87 ± 0.03 |
|  | 13 | 9 | 3.15 ± 0.63 | 99.0 | 1.34 ± 0.06 | 0.53 ± 0.07 | 1.68 ± 0.08 | 1.07 ± 0.03 | 1.07 ± 0.06 | 0.87 ± 0.06 |
|  | 3 | 17 | 4.2 ± 0.65 | 89.6 | 1.32 ± 0.07 | 0.55 ± 0.01 | 1.62 ± 0.05 | 0.98 ± 0.07 | 1.08 ± 0.09 | 0.88 ± 0.03 |
|  | 5 | 20 | 4.2 ± 0.65 | 87.2 | 1.33 ± 0.02 | 0.47 ± 0.01 | 1.53 ± 0.04 | 0.80 ± 0.04 | 1.13 ± 0.01 | 0.93 ± 0.04 |
|  | 18 | 25 | N.A | N.A. | N.A. | N.A. | N.A. | N.A. | N.A. | N.A. |
|  | 4 | 25 | 3.84 ± 0.38 | 75.4 | 1.17 ± 0.06 | 0.34 ± 0.09 | 1.43 ± 0.06 | 0.71 ± 0.04 | 0.99 ± 0.06 | 0.89 ± 0.08 |
|  | 2 | 25.5 | 3.82 ± 0.9 | 67.5 | 1.15 ± 0.09 | 0.29 ± 0.03 | 1.29 ± 0.01 | 0.55 ± 0.04 | 1.01 ± 0.10 | 0.87 ± 0.03 |
|  | 20 | 28 | N.A. | N.A. | N.A. | N.A. | N.A. | N.A. | N.A. | N.A. |
|  | 11 | 29 | 4.72 ± 0.35 | 65.3 | 1.19 ± 0.03 | 0.16 ± 0.03 | 1.09 ± 0.11 | 0.20 ± 0.10 | 1.14 ± 0.02 | 0.94 ± 0.04 |
|  | 15 | 35.5 | N.A. | N.A. | N.A. | N.A. | N.A. | N.A. | N.A. | N.A. |
| **Males** | 17 | 2 | 1.04 ± 0.15 | 87.2 | 1.36 ± 0.07 | 0.58 ± 0.02 | 1.67 ± 0.04 | 1.05 ± 0.05 | 1.10 ± 0.08 | 0.88 ± 0.05 |
|  | 14 | 2.5 | N.A. | N.A. | N.A. | N.A. | N.A. | N.A. | N.A. | N.A. |
|  | 16 | 4 | 1.26 ± 0.15 | 88.2 | 1.37 ± 0.05 | 0.57 ± 0.08 | 1.72 ± 0.13 | 1.01 ± 0.06 | 1.12 ± 0.04 | 0.96 ± 0.14 |
|  | 9 | 6 | 1.69 ± 0.34 | 93.7 | 1.37 ± 0.03 | 0.50 ± 0.07 | 1.72 ± 0.03 | 0.95 ± 0.08 | 1.13 ± 0.01 | 1.01 ± 0.07 |
|  | 19 | 15 | N.A. | N.A. | N.A. | N.A. | N.A. | N.A. | N.A. | N.A. |
|  | 21 | 16 | N.A. | N.A. | N.A. | N.A. | N.A. | N.A. | N.A. | N.A. |

N.A. = not available

**Supplementary Table 5.** Hg isotopic composition measured in blood and milk of long-finned pilot whales. The uncertainty is reported as 2SE for three sample replicates.

|  | **Whale ID** | **Age**  **(years)** | **δ^199^Hg**  **(‰)** | **δ^200^Hg**  **(‰)** | **δ^201^Hg**  **(‰)** | **δ^202^Hg**  **(‰)** | **Δ^199^Hg**  **(‰)** | **Δ^201^Hg**  **(‰)** |
| --- | --- | --- | --- | --- | --- | --- | --- | --- |
| **Blood**  **Females** | 6 | 1 | N.A. | N.A. | N.A. | N.A. | N.A. | N.A. |
|  | 7 | 1 | N.A. | N.A. | N.A. | N.A. | N.A. | N.A. |
|  | 8 | 1 | 1.27 ± 0.05 | 0.61 ± 0.05 | 1.64 ± 0.11 | 0.98 ± 0.17 | 1.02 ± 0.06 | 0.90 ± 0.01 |
|  | 1 | 2.5 | 1.35 ± 0.08 | 0.52 ± 0.03 | 1.63 ± 0.14 | 1.01 ± 0.05 | 1.10 ± 0.08 | 0.88 ± 0.11 |
|  | 12 | 3 | N.A. | N.A. | N.A. | N.A. | N.A. | N.A. |
|  | 10 | 4 | 1.36 ± 0.11 | 0.57 ± 0.02 | 1.75 ± 0.10 | 1.15 ± 0.04 | 1.07 ± 0.10 | 0.88 ± 0.07 |
|  | 13 | 9 | 1.35 ± 0.12 | 0.69 ± 0.12 | 1.82 ± 0.06 | 1.18 ± 0.07 | 1.05 ± 0.13 | 0.93 ± 0.08 |
|  | 3 | 17 | 1.43 ± 0.15 | 0.71 ± 0.13 | 1.80 ± 0.12 | 1.27 ± 0.15 | 1.11 ± 0.14 | 0.84 ± 0.04 |
|  | 5 | 20 | 1.44 ± 0.05 | 0.61 ±0.10 | 1.78 ± 0.06 | 1.10 ± 0.11 | 1.16 ± 0.06 | 0.95 ± 0.08 |
|  | 18 | 25 | N.A. | N.A. | N.A. | N.A. | N.A. | N.A. |
|  | 4 | 25 | 1.37 ± 0.06 | 0.59 ±0.05 | 1.70 ± 0.05 | 1.04 ± 0.02 | 1.11 ± 0.06 | 0.92 ± 0.07 |
|  | 2 | 25.5 | 1.31 ± 0.03 | 0.57 ± 0.06 | 1.68 ± 0.03 | 0.99 ± 0.03 | 1.06 ± 0.03 | 0.94 ± 0.01 |
|  | 20 | 28 | N.A. | N.A. | N.A. | N.A. | N.A. | N.A. |
|  | 11 | 29 | 1.42 ± 0.06 | 0.53 ± 0.06 | 1.69 ± 0.01 | 0.97 ± 0.11 | 1.17 ± 0.06 | 0.96 ± 0.08 |
|  | 15 | 35.5 | 1.36 ± 0.05 | 0.47 ± 0.02 | 1.67 ± 0.09 | 0.90 ± 0.06 | 1.14 ± 0.05 | 0.99 ± 0.06 |
| **Blood**  **Males** | 17 | 2 | 1.39 ± 0.05 | 0.61 ±0.09 | 1.68 ± 0.10 | 1.03 ± 0.09 | 1.13 ± 0.04 | 0.91 ± 0.05 |
|  | 14 | 2.5 | 1.41 ± 0.08 | 0.67 ± 0.06 | 1.85 ± 0.11 | 1.23 ±0.08 | 1.10 ± 0.06 | 0.93 ± 0.06 |
|  | 16 | 4 | 1.38 ± 0.05 | 0.59 ± 0.09 | 1.70 ± 0.06 | 1.07 ± 0.08 | 1.12 ± 0.04 | 0.96 ± 0.05 |
|  | 9 | 6 | 1.35 ± 0.06 | 0.56 ± 0.08 | 1.65 ± 0.08 | 0.96 ± 0.15 | 1.11 ± 0.03 | 0.93 ± 0.04 |
|  | 19 | 15 | N.A. | N.A. | N.A. | N.A. | N.A. | N.A. |
|  | 21 | 16 | N.A. | N.A. | N.A. | N.A. | N.A. | N.A. |
| **Milk** | 11 | 29 | 1.18 ± 0.03 | 0.14 ± 0.05 | 1.06 ± 0.04 | 0.12 ± 0.04 | 1.15 ± 0.04 | 0.97 ± 0.04 |

N.A. = not available

**Supplementary Table 6.** Hg isotopic composition measured in the isolated HgSe nanoparticles from different tissues. The uncertainty is reported as 2SE for three sample replicates.

|  | **Whale ID** | **Age**  **(years)** | **δ^199^Hg**  **(‰)** | **δ^200^Hg**  **(‰)** | **δ^201^Hg**  **(‰)** | **δ^202^Hg**  **(‰)** | **Δ^199^Hg**  **(‰)** | **Δ^201^Hg**  **(‰)** |
| --- | --- | --- | --- | --- | --- | --- | --- | --- |
|  | 14 | 2.5 | 0.68 ± 0.09 | -0.38 ± 0.04 | 0.07 ± 0.03 | -0.85 ± 0.06 | 0.89 ± 0.09 | 0.70 ± 0.02 |
|  | 13 | 9 | 0.75 ± 0.09 | -0.32 ± 0.06 | 0.20 ± 0.08 | -0.74 ± 0.03 | 0.94 ± 0.08 | 0.76 ± 0.06 |
| **Liver** | 3 | 17 | 0.77 ± 0.11 | -0.18 ± 0.08 | 0.38 ± 0.10 | -0.43 ± 0.13 | 0.88 ± 0.10 | 0.71 ± 0.06 |
|  | 2 | 25.5 | 0.87 ± 0.07 | -0.05 ± 0.05 | 0.71 ± 0.03 | -0.14 ± 0.10 | 0.91 ± 0.08 | 0.81 ± 0.09 |
|  | 11 | 29 | 0.84 ± 0.07 | -0.01 ± 0.07 | 0.70 ± 0.04 | -0.09 ± 0.07 | 0.86 ± 0.07 | 0.76 ± 0.08 |
| **Muscle** | 2 | 25.5 | 1.03 ± 0.03 | 0.11 ± 0.05 | 0.91 ± 0.07 | 0.18 ± 0.11 | 0.99 ± 0.03 | 0.78 ± 0.07 |
|  | 11 | 29 | 0.94 ± 0.13 | 0.00 ± 0.08 | 0.76 ± 0.04 | -0.01 ± 0.18 | 0.95 ± 0.17 | 0.77 ± 0.10 |

**Supplementary Table 7.** Microwave program used for microwave-assisted acid digestion in a Milestone Ethos One High-Performance Microwave Digestion System.

| **Step** | **Temperature (°C)** | **Time (min)** |
| --- | --- | --- |
| 1 | Room temperature to 70 | 5 |
| 2 | 70 to 90 | 7 |
| 3 | 90 | 5 |
| 4 | 90 to 120 | 7 |
| 5 | 120 | 5 |
| 6 | 120 to 150 | 7 |
| 7 | 150 | 5 |

**Supplementary Table 8.** Recoveries and mercury isotope ratio data for reference materials used for QA/QC. The uncertainty of the in-house standard is reported as 2SD because it is considered the external reproducibility of the method. For the other materials, the uncertainty is reported as 2SE for the different replicate measurements.^1^

|  | | **n** | | | **Recovery THg**  **(%)** | | **Recovery MeHg**  **(%)** | |
| --- | --- | --- | --- | --- | --- | --- | --- | --- |
| **BCR CRM 464** | | 3 | | | 101 ± 6 | | --- | |
| **NRC-CNRC DORM - 4** | | 3 | | | 98 ± 5 | | --- | |
| **NRC-CNRC TORT - 3** | | 3 | | | 98 ± 5 | | --- | |
| **NRC-CNRC DOLT - 2** | | 3 | | | --- | | 103 ± 6 | |
| **NRC-CNRC DORM - 2** | | 3 | | | --- | | 102 ± 8 | |
|  |  | | **n** | **δ^202^Hg**  **(‰)** | | **Δ^199^Hg**  **(‰)** | | **Δ^201^Hg**  **(‰)** |
| **In-house standard** |  | | 147 | -0.59 ± 0.12 | | 0.00 ± 0.12 | | 0.00 ± 0.12 |
| **BCR CRM 464** | This work | | 14 | 0.44 ± 0.07 | | 2.09 ± 0.05 | | 1.72 ± 0.03 |
|  | Epov *et al.*^[2](#_ENREF_1)^ | | 7 | 0.59 ± 0.08 | | 2.18 ± 0.03 | | 1.79 ± 0.03 |
| **NRC-CNRC DORM - 4** |  | | 4 | 0.32 ± 0.06 | | 1.65 ± 0.12 | | 1.26 ± 0.10 |
| **NRC-CNRC TORT - 3** |  | | 3 | -0.18 ± 0.05 | | 0.54 ± 0.01 | | 0.51 ± 0.10 |

**Supplementary Figure 1.** Three-isotope plots: δ^199^Hg (A), δ^200^Hg (B) and δ^201^Hg (C) *vs* δ^202^Hg for all the samples analyzed in this work. The error bars are the SD of the average of all samples for each of the different tissues and biofluids.

**Supplementary Figure 2.** δ^202^Hg as a function of % MeHg for liver (black squares) and muscle (blue triangles) tissues. Generally, δ^202^Hg values increase as a function of the MeHg fraction, except for livers with the lowest % MeHg. Uncertainty expressed as 2SD of the in-house standard.

**Supplementary Figure 3.** Δ^199^Hg *vs* δ^202^Hg values obtained for all tissue samples and biological fluids analyzed in this work. The error bars are the SD of the average of all samples for each of the different tissues and biofluids.

**Supplementary Figure 4.** Δ^199^Hg *vs* Δ^201^Hg values obtained for all the samples analyzed in this work. The line represents the average Δ^199^Hg/Δ^201^Hg ratio (1.21).The uncertainty is the external reproducibility of the in-house standard.

**Supplementary Figure 5.** δ^202^Hg (black – left y-axis) and MeHg fraction (% MeHg, red – right y-axis) obtained for kidney as a function of age. The uncertainty is the external reproducibility of the in-house standard.

**Supplementary References**

1 Blum, J. D. & Bergquist, B. A. Reporting of variations in the natural isotopic composition of mercury. Anal. Bioanal. Chem. 388, 353 - 359 (2007).

2 Epov, V. N. et al. Simultaneous Determination of Species Specific Isotopic Composition of Hg by Gas Chromatography Coupled to Multicollector ICPMS. *Anal. Chem*. **80**, 3530 – 3538 (2008).
